# Three-dimensional changes in the upper airway and craniomaxillofacial morphology of patients with Angle Class III malocclusion treated with a Frankel III appliance

**DOI:** 10.1186/s12903-021-02013-0

**Published:** 2021-12-09

**Authors:** Yinan Liu, Kai Yang

**Affiliations:** Department of Orthodontics, Beijing Stomatological Hospital, Capital Medical University, Tian Tan Xi Li #4, Dongcheng District, Beijing, 100050 China

**Keywords:** Frankel III appliance, Upper airway, Craniomaxillofacial morphology

## Abstract

**Background:**

Angle Class III malocclusion, characterized by a concave profile, can cause serious harm to children’s physical and mental health. The Frankel III appliance is an effective treatment for Angle Class III malocclusion in mixed denition. We explored three-dimensional changes in the upper airway and craniomaxillofacial morphology, after one year of Frankel III appliance treatment, in children with Angle Class III malocclusion.

**Methods:**

We included 20 children (9 males), aged 8–10 years, with Angle Class III malocclusion from the Orthodontics Department of our hospital. Each child was treated with a Frankel III appliance for one year. Cone beam computed tomography was performed before and after treatment to evaluate three-dimensional changes in the upper airway and craniomaxillofacial morphology.

**Results:**

After one year of treatment, in the upper airway, we observed significant increases in the nasopharynx volume and height (*P* < 0.05); the velopharyngeal volume, height, and average cross-sectional area (*P* < 0.05); the glossopharynx volume and minimum cross-sectional area (*P* < 0.05); and the laryngopharynx height (*P* < 0.05). Accordingly, the total upper airway volume, height, and average cross sectional area increased significantly (*P* < 0.05). An examination of craniomaxillofacial morphology showed significant increases in some bone tissues (*P* < 0.05) and dental measurements, and a significant reduction in the inclination of the mandibular central incisor (*P* < 0.05).

**Conclusion:**

Children with Angle class III malocclusion treated with a Frankel III appliance showed no upper airway narrowing, even after repositioning the mandible posteriorly. Moreover, treatment promoted forward maxilla development and increased its width, in both the dental arch and alveolar bone, which provided a more harmonious craniofacial morphology.

## Background

Angle Class III malocclusion has long been considered a complicated maxillofacial disorder characterized by a concave profile, which may include mandibular protrusion, maxillary retrusion, or a combination of the two [1]. If the symptoms worsen with patient growth, the condition might require orthognathic surgery in adulthood. Thus, orthodontists should place more emphasis on the early treatment of Angle Class III malocclusion.

The Frankel III appliance is an effective treatment for Angle Class III malocclusion that is currently implemented worldwide [2]. Before applying the Frankel III appliance, the mandible was gently guided posteriorly, to the centric position, for wax bite construction [3]. The Frankel III appliance treatment led to an occlusal plane rotation that shifted the molar configuration from a Class III to a Class I angle [4].

Some studies have reported that treatments with a Frankel III appliance had clear effects on maxillary development [5], dentoalveolar development, skeletal widths [6], and the shape and position of the mandible and maxilla [5]. However, they mentioned that analyses based on two-dimensional cephalometric measurements as the sole indicators had some shortcomings, which may have affected the results. Moreover, upper airway is closely related to the craniomaxillofacial structure [7, 8]. But to the best of our knowledge, no previous studies have described changes in pharyngeal size after treating Angle class III malocclusions with Frankel III appliance in children that are growing and developing.

Therefore, the objective of study is to explore the three-dimensional changes in the upper airway and craniomaxillofacial morphology after treatment with a Frankel III appliance using the cone beam computed tomography (CBCT).

## Materials and method

### Study design and sample

This study included 20 children (9 males and 11 females), 8 to 10 years old, in a stage of mixed dentition. Patients were recruited from the Orthodontics Department of our hospital. The selection criteria were Angle Class III malocclusion; ANB < 0°; an anterior crossbite, where the edge-to-edge incisor relationship was in a retruded contact position; good cooperation during the treatment period; the patient had undergone one year of orthodontic treatment with a Frankel III appliance; CBCTs were taken before and after treatment, and image areas were complete and clear, without movement artifacts; and the images had to include clear views of the nasion point (N), hyoid bone, epiglottis valley bottom, and other points of interest.

### Data collection

All CBCT scans were performed by a specialist in radiology in the Radiology Department of our hospital. Under the unified mode, the CBCT equipment (NewTom VG; AFP; Verona, Italy) was set to the following parameters: 1–20 mA (pulsed mode), 110 kV voltage, 3.6 s effective exposure time, 60 s reconstruction time, whole skull mode. During image acquisition, patients were instructed to bite down, in the intercuspal position. All CBCT images were exported in DICOM format (Digital Imaging and Communications in Medicine), and three-dimensional images of the craniomaxillofacial morphology, upper airway, and hyoid were reconstructed with Dolphin Imaging software (version 11.8; Dolphin Imaging & Management Solutions, Chatsworth, CA).

### Data analysis

**Fig. 1:**
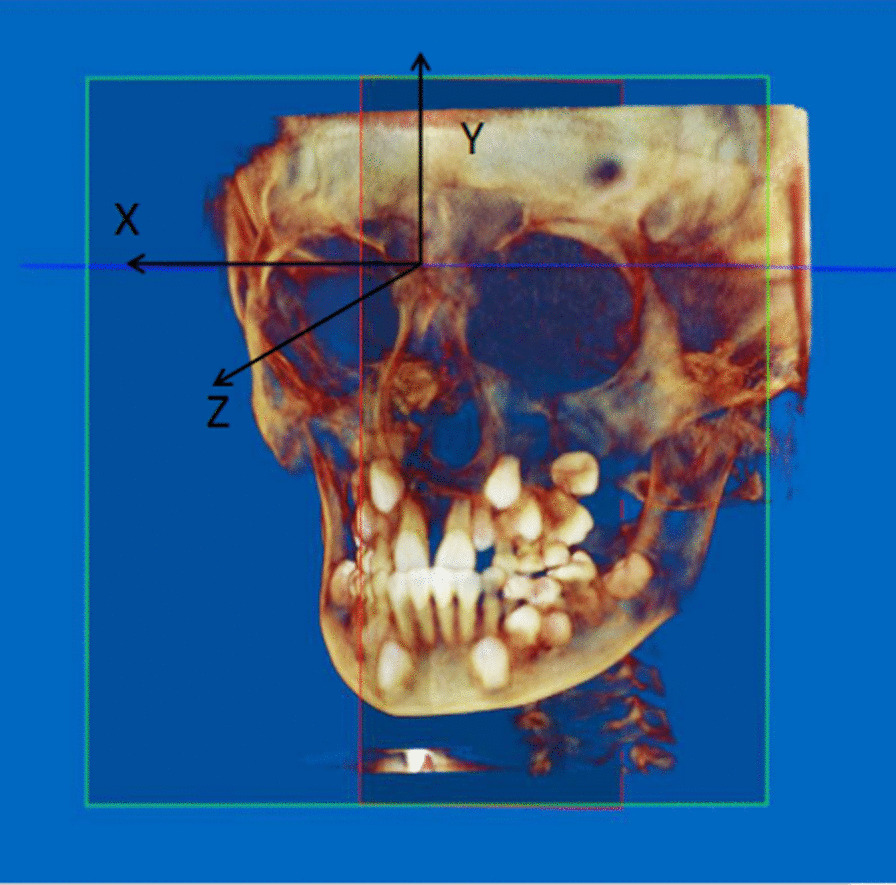
Determination of the three-dimensional coordinate system

Before taking any measurements, the three-dimensional coordinate system was established. The orientation function provided in the software was applied to adjust the head position and orient the planes in the three-dimensional coordinate system (Fig. 1). Referring to the methods of Shin et al. [9], Yang et al. [7], and the three-dimensional coordinate axis instructions provided in the software, we established the nasion point as the origin in the three-dimensional coordinate system.

**Fig. 2:**
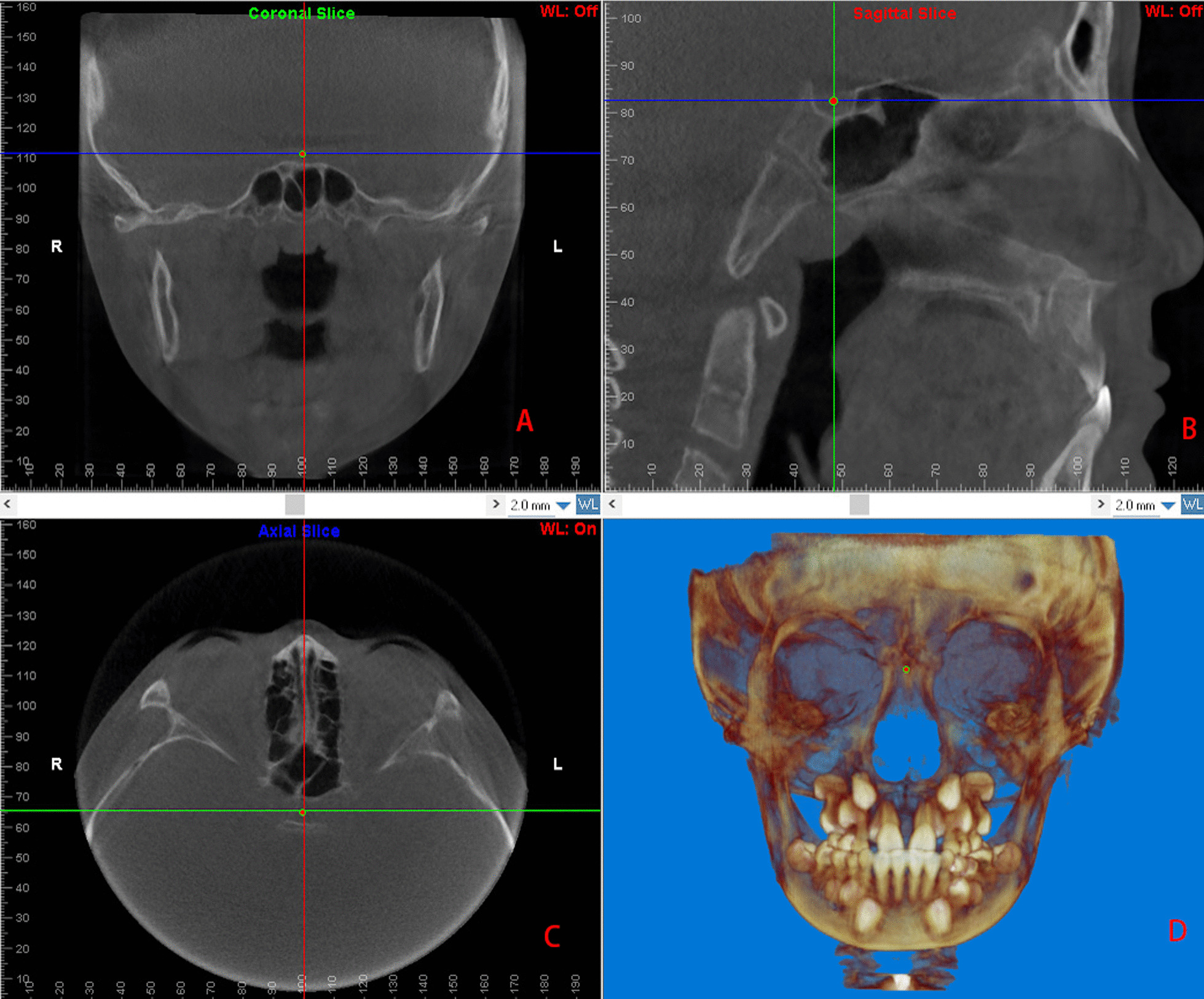
Three-dimensional fixed points. This figure shows the sella point on the coronal plane (**A**), sagittal plane (**B**), horizontal plane (**C**), and its overall view (**D**)

First, we set the Frankfort horizontal plane, which was aligned with the top point of the external auditory canal and the lowest point of the infraorbital edge. When the bilateral orbitales and porions were not located on the same plane, we set the Frankfort horizontal plane between them, at the minimum squared distance from all four points. Then, we determined the midsagittal plane, which was aligned with the nasion point, the anterior nasal spine (ANS) point, and the basion point. The X-axis was defined as the line parallel to the Frankfort horizontal plane that passed through the nasion point. The Z-axis was defined as the line perpendicular to the X-axis that passed through the nasion point on the midsagittal plane. The Y-axis was defined as the line perpendicular to the Z- and X-axes that passed through the nasion point. Therefore, any point in this space was automatically fixed in three-dimensional coordinate values (x, y, z) with the software (Fig. 2). The craniomaxillofacial morphology assessment was based on 25 landmarks (Table 1), each measured on the X-, Y-, and Z-axes.

**Table 1:** Items measured to assess craniomaxillofacial morphology

Items	Definition
SNA	The angle formed by lines between the sella, nasion, and subspinale points, which represents the sagittal position of the maxilla
SNB	The angle formed by lines between the sella, nasion, and supramental points, which represents the sagittal position of the mandible
ANB	The angle formed by lines between the subspinale, nasion, and supramental points, which represents the sagittal position of the maxilla and mandible
S–N	The distance between the sella and nasion points, which indicates the length of the anterior basis cranii
S-Ba	The distance between the sella and basion points, which indicates the length of posterior basis cranii
ZyR-ZyL	The distance between the bilateral zygomatic arch which indicates the width of the hard tissue surface
N-Me	The distance between the nasion and menton points, which indicates the overall height of the hard tissue
ANS-Me	The distance between the anterior nasal spine (ANS) and the menton point, which indicates the height of the lower hard tissue
ANS-Me/N-Me	The ratio of the ANS-Me to the N-Me
PNS-ANS	The distance between the anterior nasal spine (ANS) and the posterior nasal spine (PNS)
J-J	The distance between the bilateral jugal points (the most concave point between the maxillary tubercle and the zygomatic process), which indicates the width of the maxilla
GoR-GoL	The distance between the bilateral gonion points, which indicates the width of the mandible
Co-Go	The distance between the condylion and gonion points, which indicates the length of the ramus of the mandible
Go-Me	The distance between the gonion and menton points, which indicates the length of the mandible
Co-Go-Me	The angle formed by lines between the condylion, gonion, and menton points, which indicates the angle of the mandible
MP-FH	The angle between the plane of mandible inclination (MP) and the Frankfort horizontal (FH) plane, which indicates the angle of the mandible
U1-SN	The angle between the long axis of the upper incisor (U1) and the sella-nasion (SN) plane of the maxillary central incisor, which indicates the inclination of the maxillary central incisor
L1-MP	The angle between the long axis (L1) of the mandible central incisor and its plane of inclination (MP)
U1-L1	The angle of upper incisor to lower incisor
MBBW (maxillary buccal basal bone width)	The horizontal distance between the most concave points of the buccal basal bone, near the root tip, on the coronal plane
MFMW (maxillary first molar width)	The horizontal distance between the central fossa of the first molar on both sides of the maxilla, which indicates the width of the posterior arch
H-CVP	The distance between the topmost point of the hyoid body (H) and a line placed tangent to the anterior surfaces of the bodies of the second, third, and fourth cervical vertebrae
H-MP	The distance between the uppermost point of the hyoid body and the MP
H-FH	The distance between the topmost point of the hyoid body and the Frankfort plane
PNS-CVP	The distance between the posterior nasal spine (PNS) and a line placed tangent to the anterior surfaces of the bodies of the second, third, and fourth cervical vertebrae
Me-CVP	The distance between the menton and a line placed tangent to the anterior surfaces of the bodies of the second, third, and fourth cervical vertebrae

**Fig. 3:**
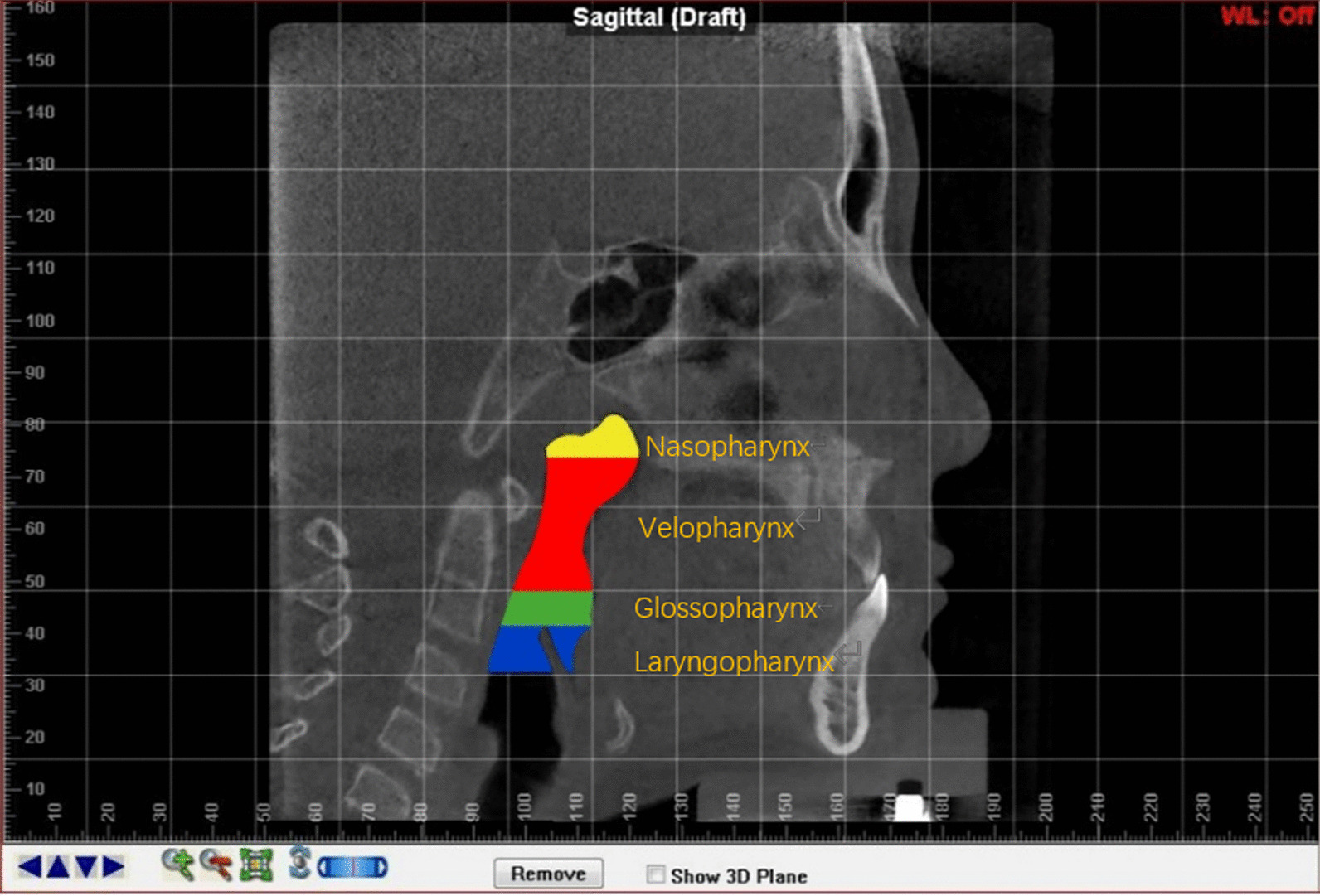
Sagittal view of the upper airway anatomical subsections

**Fig. 4:**
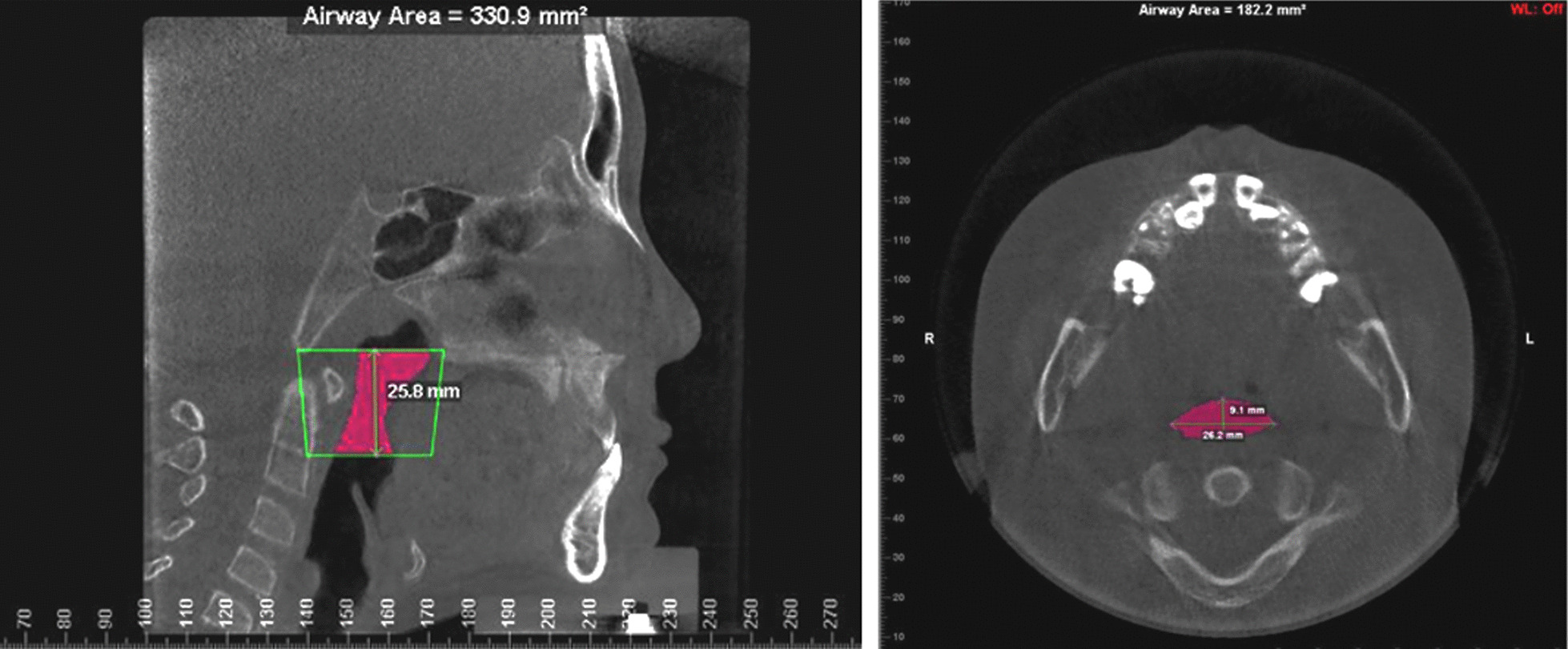
Calculate the upper airway items. The volume is calculated as (left) the height of the pharyngeal cavity, multiplied by (right) the minimum cross-sectional area, which is automatically

The upper airway sections were based on the anatomical divisions described by Yang et al. [7], and Shin et al. [9]. In this study, the nasopharynx area was located between the top of the upper airway and the horizontal plane that passed through the posterior nasal spine. The velopharynx area was located between the end of the nasopharynx and the horizontal plane that passed through the end of the soft palate. The glossopharynx area was between the end of the velopharynx and the horizontal plane that passed through the top of the epiglottis. Finally, the laryngopharynx area was between the end of the glossopharynx and the horizontal plane that passed through the epiglottis vallecula (Fig. 3). The total upper airway area was defined as sum of these four sections. The software could automatically calculate the airway volume, find the minimum cross-section, and calculate the minimum cross-sectional area (Fig. 4). To evaluate the three-dimensional changes in the upper airway, we measured the volumes, heights, and diameters. Additionally, we calculated the average cross-sectional areas of the four pharyngeal cavities (Table 2).

**Table 2:** Items measured to assess the upper airway

Items	Definition
Nasopharyngeal volume (Na-V)	Nasopharynx volume, calculated by simulation
Nasopharyngeal height (Na-H)	The linear distance between the upper and lower boundaries of the nasopharynx
Nasopharyngeal average cross-sectional area (Na-CSAavg)	Ratio of the nasopharyngeal volume to its height
Velopharyngeal volume (Ve-V)	Velopharyngeal volume, calculated by simulation
Minimum velopharyngeal cross-sectional area (Ve-CSAmin)	The minimum cross-sectional area of the palatopharyngeal segment, identified and calculated automatically
Anteroposterior velopharyngeal diameter (Ve-AP)	Anterior–posterior distance at the minimum cross-section of the velopharyngeal segment
Lateral velopharyngeal diameter (Ve-LAT)	Lateral-medial distance at the minimum cross-section of the velopharyngeal segment
Velopharyngeal height (Ve-H)	Distance between the upper and lower boundaries of the velopharyngeal segment
Velopharyngeal average cross-sectional area (Ve-CSAavg)	Ratio of the velopharyngeal volume to its height
Glossopharyngeal volume (Gl-V)	Glossopharyngeal volume, calculated automatically by the system
Glossopharyngeal minimum cross-sectional area (Gl-CSAmin)	The minimum cross-sectional area of the glossopharyngeal segment, identified and calculated automatically
Anteroposterior glossopharyngeal diameter (Gl-AP)	Anterior–posterior distance at the minimum cross-section of glossopharyngeal segment
Lateral glossopharyngeal diameter (Gl-LAT)	Lateral-medial distance at the minimum cross-section of glossopharyngeal segment
Glossopharyngeal height (Gl-H)	The linear distance between the upper and lower boundaries of the glossopharyngeal segment
Laryngopharynx volume (La-V)	Laryngopharynx volume, calculated by simulation
Minimum laryngopharynx cross-sectional area (La-CSAmin)	The minimum cross-sectional area of the laryngopharynx segment, identified and calculated automatically
Anteroposterior laryngopharynx diameter (La-AP)	Anterior–posterior distance at the minimum cross-section of the laryngopharyngeal segment
Lateral laryngopharynx diameter (La-LAT)	Lateral-medial distance at the minimum cross-section of the laryngopharyngeal segment
Laryngopharynx height (La-H)	The linear distance between the upper and lower boundaries of the laryngopharyngeal segment
Laryngopharynx average cross-sectional area (La-CSAavg)	Ratio of the laryngopharynx volume to its height
Total volume of the upper airway (T-V)	Sum of the volumes of the upper airway segments
Total height of the upper airway (T-H)	Sum of the heights of the upper airway segments
Average cross-sectional area of the upper airway (T-CSAavg)	Ratio of the total volume of the upper airway to its total height

### Statistical analysis

To reduce the errors, all the values were measured three times by an orthodontic student with professional training, and the average values were analyzed. We performed statistical analyses with SPSS software (version 22.0; IBM, Armonk, NY). We performed the Wilcoxon signed rank test to evaluate differences between measurements taken before and after treatment.

## Results

This prospective study included 20 patients, aged 8–10 years. The three-dimensional changes observed in the upper airway are shown in Table 3. After one year of treatment with the Frankel III appliance, we observed significant increases in the height and volume of the nasopharynx (*P* < 0.05); the height, volume, and the average cross-sectional area of the velopharynx (*P* < 0.05); the volume and minimum cross-sectional area of the glossopharynx (*P* < 0.05); and the height of the laryngopharynx (*P* < 0.05). Accordingly, we observed significant increases in the volume, height, and average cross-sectional area of the total upper airway (*P* < 0.05). The other items in the upper airway increased, but not significantly (*P* > 0.05), except for the anteroposterior velopharyngeal diameter, which remained nearly constant. Overall, no measurement was significantly reduced in the upper airway.

**Table 3:** Three-dimensional measurements in the upper airway before and after treatment

Section	Items^a^	Before	After	*P*
Nasopharynx	Na-V (mm^3^)	3701.09 ± 1292.52	4543.21 ± 1414.34	0.003**
	Na-H (mm)	11.05 ± 1.91	12.34 ± 2.45	0.001**
	Na-CSAavg (mm^2^)	343.05 ± 135.25	375.72 ± 128.38	0.079
Velopharynx	Ve-V (mm^3^)	6209.95 ± 2578.33	7735.10 ± 3173.06	0.009**
	Ve-CSAmin (mm^2^)	131.21 ± 78.71	166.99 ± 105.15	0.067
	Ve-AP (mm)	12.08 ± 3.15	12.02 ± 3.54	0.970
	Ve-LAT (mm)	18.58 ± 6.86	20.54 ± 7.01	0.232
	Ve-H (mm)	24.74 ± 2.80	26.26 ± 3.48	0.004**
	Ve-CSAavg (mm)	253.41 ± 104.99	295.58 ± 115.92	0.017*
Glossopharynx	Gl-V (mm^3^)	2831.44 ± 1449.25	3859.90 ± 2248.44	0.021*
	Gl-CSAmin (mm^2^)	139.56 ± 65.01	177.12 ± 75.66	0.025*
	Gl-AP (mm)	11.43 ± 2.82	12.42 ± 2.76	0.185
	Gl-LAT (mm)	21.71 ± 7.50	23.341 ± 7.75	0.467
	Gl-H (mm)	13.17 ± 3.85	14.52 ± 3.95	0.370
	Gl-CSAavg (mm^2^)	217.84 ± 85.48	260.49 ± 113.70	0.086
Laryngopharynx	La-V (mm^3^)	2485.25 ± 995.76	3283.71 ± 2120.79	0.057
	La-CSAmin (mm^2^)	156.05 ± 74.19	176.49 ± 85.46	0.526
	La-AP (mm)	9.94 ± 3.07	11.01 ± 3.56	0.263
	La-LAT (mm)	28.47 ± 3.24	29.37 ± 4.89	0.191
	La-H (mm)	9.40 ± 1.58	10.39 ± 2.27	0.017*
	La-CSAavg (mm^2^)	259.84 ± 82.35	302.68 ± 132.98	0.135
Total upper airway	T-V (mm^3^)	15,227.72 ± 5235.83	19,421.92 ± 7102.11	0.005**
	T-H (mm)	58.356 ± 5.37	63.51 ± 6.88	0.000**
	T-CSAavg (mm^2^)	262.25 ± 87.59	303.64 ± 95.74	0.025*

**P < 0.01, *P < 0.05

^a^See Table 2 for definitions of these abbreviations

The changes in craniomaxillofacial morphology are presented in Table 4. After one year of treatment with the Frankel III appliance, we observed large changes in craniomaxillofacial morphology. In the sagittal direction, SNA and ANB increased significantly (*P* < 0.05), which indicated that the Frankel III appliance promoted the development of the maxilla and adjusted the relationship between the two jaws. We also observed significant changes in the sella-nasion (S-N) and sella-basion (S-Ba) distances (*P* < 0.05). We observed significant increases in the distance between the nasion and menton points (N-Me), the distance between the ANS and the menton (ANS-Me), and the ratio of the ANS-Me to the N-Me (*P* < 0.05), which indicated an increase in the anterior face height, particularly in the anterior lower face. We also observed an increase in the distance between the anterior and posterior nasal spines (PNS-ANS) (*P* < 0.05), which indicated that the presenting length of the maxilla increased. The distance between the condylion and gonion points (Co-Go) increased, which indicated an increase in the length of the ramus of the mandible (*P* < 0.05). We also observed significant increases in the distance between the gonion and menton points (Go-Me) and the distance between the menton point and the cervical vertebrae plane (Me-CVP), which indicated an increase in the length of the mandible (*P* < 0.05). The significant increases observed in the distance between the topmost point of the hyoid body and the cervical vertebrae plane (H-CVP) and the distance between the topmost point of the hyoid body and the Frankfort plane (H-FH) indicated that the hyoid bone had moved forward and downward (*P* < 0.05) with treatment. The inclination of the maxillary central incisor (U1-SN) increased significantly, and the angle between the long axis of the mandible central incisor and its plane of inclination (L1-MP) decreased significantly (*P* < 0.05). These findings indicated that the upper anterior teeth had inclined labially, and the lower anterior teeth had inclined lingually.

**Table 4:** Three-dimensional measurements of craniomaxillofacial morphology before and after treatment

Measurement items^a^	Before	After	*P*
SNA (°)	79.73 ± 2.74	81.47 ± 2.94	0.001**
SNB (°)	80.15 ± 2.93	79.40 ± 3.42	0.076
ANB (°)	− 0.42 ± 2.57	2.07 ± 1.94	0.000**
S–N (mm)	59.27 ± 3.05	60.62 ± 3.58	0.000**
S-Ba (mm)	41.55 ± 2.10	43.14 ± 3.16	0.000**
ZyR-ZyL (mm)	109.11 ± 3.23	111.18 ± 3.19	0.004**
N-Me (mm)	101.74 ± 11.70	109.44 ± 6.24	0.000**
ANS-Me (mm)	57.47 ± 3.17	62.05 ± 3.89	0.000**
ANS-Me/N-Me (%)	48.77 ± 2.29	50.31 ± 2.17	0.019*
PNS-ANS (mm)	42.80 ± 3.92	45.27 ± 4.26	0.001**
J-J (mm)	67.49 ± 2.47	70.63 ± 4.06	0.000**
GoR-GoL (mm)	82.52 ± 3.25	84.48 ± 4.27	0.003**
Co-Go (mm)	52.24 ± 4.75	55.65 ± 4.21	0.003**
Go-Me (mm)	69.79 ± 5.49	73.14 ± 6.07	0.021*
Co-Go-Me (mm)	126.55 ± 5.68	125.29 ± 6.04	0.370
MP-FH (mm)	33.38 ± 5.26	34.60 ± 5.18	0.232
U1-SN (°)	102.11 ± 7.98	107.24 ± 4.62	0.007**
L1-MP (°)	108.74 ± 17.51	105.21 ± 17.15	0.005**
U1-L1 (°)	131.15 ± 8.71	130.60 ± 6.56	0.723
MBBW (mm)	63.42 ± 2.36	64.87 ± 2.22	0.001**
MFMW (mm)	48.81 ± 2.38	50.70 ± 2.68	0.000**
H-CVP (mm)	28.035 ± 3.00	30.48 ± 4.76	0.008**
H-MP (mm)	24.34 ± 3.49	25.06 ± 3.87	0.239
H-FH (mm)	82.79 ± 5.21	87.28 ± 7.59	0.001**
PNS-CVP (mm)	22.40 ± 4.60	22.17 ± 4.38	0.550
Me-CVP (mm)	64.13 ± 6.44	67.72 ± 8.57	0.040*

**P < 0.01, *P < 0.05

^a^See Table 1 for definitions of these abbreviations

The distance between the bilateral jugal points (J-J) and the distance between the bilateral gonion points (GoR-GoL) increased with treatment, which indicated that the widths of the maxilla and mandible increased significantly (*P* < 0.05). We also observed significant increases in the maxillary first molar width (MFMW) and the maxillary buccal basal bone width (MBBW) (*P* < 0.05), which indicated that the widths of the maxilla arch and base bone had increased with treatment.

## Discussion

In this study, SNA angle significantly increased, by 1.74°, after one year of treatment. This finding verified the effects of Frankel III appliance treatment on the maxilla. Besides, the maxilla widths in both the dental and alveolar areas increased significantly. These increases may have been caused by the vestibular shields of the Frankel III device, which eliminated the restrictive pressure of the buccinator on the maxilla bone and dental arch. Additionally, we observed a significant change in the length of the ramus of the mandible and the distance between the menton and the cervical vertebrae. This finding indicated that the growth of mandible was not inhibited. Overall, the total volume, height, and average cross-sectional area of the upper airway increased significantly. Thus, the total upper airway was larger after treatment than before treatment with the Frankel III appliance.

The nasopharynx is one of the most important areas of the upper airway, due to its close relationship to the occurrence of obstructive sleep apnea hypopnea syndrome [10]. Brodie and King stated that the total depth of the nasopharynx is established in the first or second years of life [11, 12]. King also showed that, with growth, increases in the depth of the nasopharynx at the spheno-occipital junction are minimized by the forward growth of the anterior arch of the atlas. Furthermore, there is a positive correlation between the cranial base and the nasopharyngeal depth; thus, the more obtuse the base, the greater the depth, as mentioned by Ricketts and Bergland [10, 11]. In contrast to the early establishment of the nasopharyngeal depth, King demonstrated that the nasopharyngeal height continued to increase until maturity [12]. He accounted for this increase by the descent of the hard palate and cervical vertebrae from the cranium. Bergland found that nasopharyngeal height increased by 38% [13], from six years of age to maturity. Similarly, in our study, during the one year of treatment, the nasopharynx height increased by 11.67%, and its volume increased by 22.75%. However, the average cross-section of nasopharynx did not increase significantly, which may be due to the fact that the nasopharynx depth had been determined in early childhood.

It also has been reported that there was a countless relationship between the positions of the maxilla and nasopharynx [14, 15]. Indeed, maxillary protraction significantly increased the dimensions of both the naso- and oro-pharyngeal airways. In our study, the distance between the posterior nasal spine and the cervical spine (PNS-CVP, Table 4) did not significantly change, which indicated that the distance from the back of the maxilla to the upper airway did not change. Consequently, there was no significant change in the depth of the nasopharynx. In other words, the Frankel III appliance increased the development of the anterior maxilla without changing the posterior maxilla.

The velopharynx is a muscular valve that extends from the posterior surface of the hard palate (roof of the mouth) to the posterior pharyngeal wall [16]. The velopharynx and surrounding oral and pharyngeal structures change rapidly during early development.

The velum and epiglottis separate at about 4 to 6 months of age [17], as the larynx moves from the level of the first cervical vertebra to the level of the third cervical vertebra. The rate of laryngeal descent is accelerated during the first 2 years of life, when the pharyngeal length increases by up to 2 cm; this growth period is followed by more gradual lengthening [18]. This movement is accompanied by rapid growth of the pharynx, in the vertical dimension, from its newborn length, of about 4 cm to its adult length of approximately 12 cm [19]. In contrast, the anteroposterior dimension of the pharynx changes little from infancy to adulthood [12, 13, 18, 20, 21]. In our study, we observed clear increases in the height (6.14%) and volume (24.56%) of the velopharynx, during the one year of treatment. This finding indicated that increases in the velopharyngeal height and its average cross-sectional area (Ve-CSAavg) during this period led to a corresponding increase in its volume (Ve-V). However, the velopharyngeal anterior–posterior diameter (Ve-AP) remained almost unchanged, consistent with the above study, which showed that little change occurred in the anteroposterior dimension of velopharynx.

Few previous studies have investigated the development of the glossopharynx. As a part of oropharynx, the glossopharynx develops mainly in the vertical direction [22] and mainly due to increases in the height of the cervical vertebra. This process continues until adulthood, and two rapid growth periods are observed: one at 5–7 years old and the other at 12–15 years old. In our study, the glossopharynx height also increased, although not significantly. Previous studies also showed that the hyoid position could affect the size of the upper airway. Indeed, hyoid retrogression, caused by mandible retrogression, could cause narrowing of the upper airway [23]. In our study, although the mandible was guided backward for bite construction, SNB did not decrease significantly, due to growth. In contrast, the distances between the hyoid and the cervical vertebrae and between the hyoid and the Frankfort plane increased significantly. These findings indicated that the hyoid bone had moved forward and downward, which may have contributed to the profound increases in the minimum cross-sectional area and volume of the glossopharynx.

Lieberman et al. studied the growth and development of the laryngopharynx [24]. They found that the height of the laryngopharynx increased significantly from birth to 6–8 years old, and after that, it remained stable. King also found that, at the peak of growth and development, the depth of the laryngopharynx increased little, due to the forward movements of the hyoid and mandible [12]. In the present study, the height of the laryngopharynx increased significantly, by 10.53%. Although the hyoid bone moved forward, the laryngopharynx showed increases in the minimum cross-sectional area (La-CSAmin) and the lateral and anterior–posterior diameters (La-LAT, La-AP), but the increases were not significant.

Progress of digitization in dental fields proposes a speed of treatment planning and a reliability of results [25]. The unique feature of our study was that we performed CBCT to measure three-dimensional changes in the upper airway and craniomaxillofacial morphology to obtain more comprehensive and accurate measurements. The American Dental Association recommended the following indications for CBCT in orthodontic treatments: observation of tooth development, limitation of tooth movement, airway evaluation, craniofacial morphology etc. [26]. CBCT has several advantages over 2D imaging, such as improved image quality, three-dimensional reconstructions, and a 1:1 ratio, which allowed accurate determination of reference points [27] and reliable measurements. Moreover, CBCT provided the ability to visualize craniofacial structures with a short exposure time and a lower radiation dose, compared to traditional computed tomography [28]. Once a CBCT is acquired, there is no need for lateral, frontal, and curvilinear cephalometric radiographs, due to the three-dimensional nature of the CBCT. In our study, the two CBCTs were performed one year apart; therefore, they caused almost no radiation damage to patients.

The main limitation of this study was the lack of a control group of children with Angle Class III malocclusion. A control group might have allowed us to rule out the influence of growth and developmental factors. However, it is not ethical to follow up this disorder without taking any action.

## Conclusions

To summarize, the Frankel III appliance modified the growth and position of the mandible, which rotated backward and downward, and promoted maxillar development which produced a more harmonious relationship between the craniofacial bones. Furthermore, the upper airway did not narrow in response to this treatment.

## Data Availability

The data analyzed this article will be shared on reasonable request to the corresponding author.

## Abbreviations

CBCT: Cone beam computed tomography
DICOM: Digital Imaging and Communications in Medicine
Ve-CSAavg: Velopharyngeal average cross-sectional area
